# Evaluating Effects of Dynamic Interventions to Control COVID-19 Pandemic: A Case Study of Guangdong, China

**DOI:** 10.3390/ijerph191610154

**Published:** 2022-08-16

**Authors:** Yuan Liu, Chuyao Liao, Li Zhuo, Haiyan Tao

**Affiliations:** 1 Guangdong Provincial Engineering Research Center for Public Security and Disaster, School of Geography and Planning, Sun Yat-Sen University, Guangzhou 510006, China; 2 Southern Marine Science and Engineering Guangdong Laboratory (Zhuhai), Zhuhai 519000, China; 3 Key Laboratory of Tropical Disease Control, Ministry of Education, Sun Yat-Sen University, Guangzhou 510080, China

**Keywords:** COVID-19, dynamic intervention, efficiency evaluation, iLSEIR-DRAM model, transmission rate

## Abstract

The emergence of different virus variants, the rapidly changing epidemic, and demands for economic recovery all require continual adjustment and optimization of COVID-19 intervention policies. For the purpose, it is both important and necessary to evaluate the effectiveness of different policies already in-place, which is the basis for optimization. Although some scholars have used epidemiological models, such as susceptible-exposed-infected-removed (SEIR), to perform evaluation, they might be inaccurate because those models often ignore the time-varying nature of transmission rate. This study proposes a new scheme to evaluate the efficiency of dynamic COVID-19 interventions using a new model named as iLSEIR-DRAM. First, we improved the traditional LSEIR model by adopting a five-parameter logistic function β(t) to depict the key parameter of transmission rate. Then, we estimated the parameters by using an adaptive Markov Chain Monte Carlo (MCMC) algorithm, which combines delayed rejection and adaptive metropolis samplers (DRAM). Finally, we developed a new quantitative indicator to evaluate the efficiency of COVID-19 interventions, which is based on parameters in β(t) and considers both the decreasing degree of the transmission rate and the emerging time of the epidemic inflection point. This scheme was applied to seven cities in Guangdong Province. We found that the iLSEIR-DRAM model can retrace the COVID-19 transmission quite well, with the simulation accuracy being over 95% in all cities. The proposed indicator succeeds in evaluating the historical intervention efficiency and makes the efficiency comparable among different cities. The comparison results showed that the intervention policies implemented in Guangzhou is the most efficient, which is consistent with public awareness. The proposed scheme for efficiency evaluation in this study is easy to implement and may promote precise prevention and control of the COVID-19 epidemic.

## 1. Introduction

The coronavirus disease 2019 (COVID-19) pandemic has profoundly impacted the health, economy, and livelihood of human societies worldwide [1]. Most regions and countries have implemented various interventions to contain the spread of epidemic, such as personal measures (i.e., frequent hand hygiene, facial coverings or mask wearing, etc.), physical and social distancing measures in public spaces (i.e., adaptation or closure of schools and businesses, restrictions on public and private gatherings, staying at home, etc.), movement measures (i.e., limiting domestic and international travels, offering guidance regarding travel, etc.), special protection measures to protect special populations and vulnerable groups, and vaccination [2]. The outcomes, however, are not promising except in a few countries and regions [3]. There are many possible reasons for that, for example, virus variants, policy formulation and implementation of governments, and the response of target population to policies [4,5,6,7,8]. The current situation indicates that further improvement of current interventions is necessary, which would benefit containment of the current COVID-19 pandemic, as well as controlling other emerging infectious diseases (EIDs) for human society. Meanwhile, accurate assessment on the efficiency of interventions also becomes more and more important.

Some existing studies have evaluated the efficiency of interventions to control COVID-19 by simulating the reduction in confirmed or fatal cases under different intervention scenarios with epidemiological models [9], such as the susceptible-exposed-infected-removed (SEIR) model and its variations [10,11,12,13]. Some of these studies used individual-based models to evaluate the efficiency of interventions in micro scenarios, such as schools, refugee camps, parks, and gyms [14,15,16,17,18,19,20], while some other studies introduced asymptomatic (A), quarantine (Q), hospitalized (H), dead (D), and other processes into the original SEIR model to represent intervention effects in macro scenarios, i.e., cities, provinces, countries, and so on [11,21,22,23]. Regardless of the scales of their scenarios, these studies mainly focused on the effects of single or combined interventions at a specific intensity [12,21,24,25,26,27,28,29]. Although these studies provide certain useful results, they have two obvious limitations. First and foremost, most of the studies used a fixed transmission rate in their simulations, which may lead to bias in trend simulations due to spatio-temporal differences in the epidemic transmission [30,31,32,33]. As a result, the efficiency evaluation of COVID-19 interventions could be inaccurate and noncomparable in regions. Second, existing studies mostly applied the control variables approach in their scenario simulations, but few of them evaluated the effectiveness of different policies already applied, which, however, is more important because it provides the basis for further optimization of follow-up measures.

To address these limitations, this study proposes a scheme to evaluate the efficiency of dynamic COVID-19 interventions. Interventions refer to the various measures implemented by the government to mitigate the epidemic transmission implemented in the study area during the first wave, such as mask wearing policies, closures of schools and businesses, restrictions on public and private gatherings, domestic movement, and international travels, etc. The scheme includes three primary steps. The first step is constructing an improved logistic SEIR (iLSEIR) model, which quantifies the time-varying transmission rate with a five-parameter logistic function. The second step involves estimation of parameters in the iLSEIR model by using the DRAM algorithm, which combines delayed rejection and adaptive metropolis samplers. The third step is evaluating the efficiency of dynamic COVID-19 interventions based on a quantitative indicator, which considers both the decreasing degree of the transmission rate and the emerging time of the epidemic inflection point. In this study, we applied the scheme to seven cities in Guangdong province and compared their intervention efficiencies. We also discussed potential influencing factors.

The rest of this paper is organized as follows. Section 2 introduces the materials and methodology, including the case study area, data sources, the proposed iLSEIR-DARM model and the efficiency indicator. Section 3 presents the simulation results of the iLSEIR-DRAM, the evaluated dynamic transmission rate, and the intervention efficiency. Section 4 discusses the influencing factors of the efficiency and implications of public health. Section 5 gives the main conclusions of this study.

## 2. Materials and Methodology

### 2.1. Study Area and Data

#### 2.1.1. Study Area

**Figure 1:**
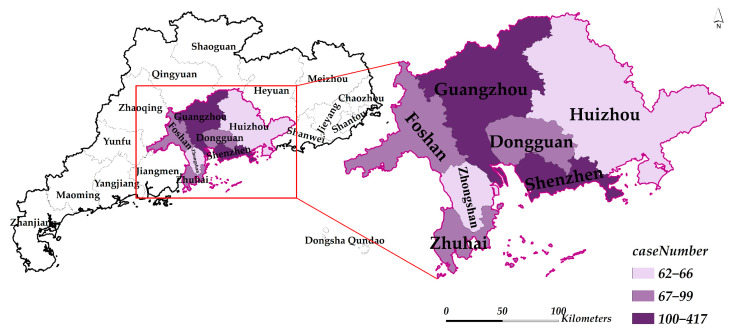
The location of seven cities in Guangdong province and their corresponding spatial distribution of the final cumulative confirmed cases during the first COVID-19 wave. For each city, the time window of the first COVID-19 wave refers to the period from 7 days before the first reported case until no new local cases for 14 consecutive days, as shown in Table 1. Additionally, the seven days considers that the epidemic in other cities has a lag of 1 to 2 weeks from Wuhan, because the infection source of other cities in the first wave was mainly imported from Wuhan [34]. The 14 days considers that the incubation period for COVID-19 generally does not exceed 14 days (http://www.gov.cn/zhengce/zhengceku/2020-02/05/content_5474791.htm) (accessed on 5 August 2022).

Since its reform and opening in the 1980s, Guangdong has developed into one of the most populous, open, innovative, and dynamic regions in China. With comprehensive transportation systems, the cities in the region have been playing important roles in linking economics at home and abroad. Large-scale movements, however, also indicate higher transmission risks of COVID-19 in these cities [8]. From 19 January 2020 to 5 March 2020, a total of 1351 confirmed cases were reported in Guangdong, which is the highest for the same period in China. In this study, we selected seven cities in Guangdong province, including Shenzhen, Guangzhou, Dongguan, Zhuhai, Foshan, Zhongshan, Huizhou, to evaluate the COVID-19 dynamic epidemic intervention efficiency (Figure 1). The cumulative confirmed cases in these cities accounted for over 85% of Guangdong Province during the first wave of the outbreak, each city with a number of cumulative confirmed cases more than 25. Other cities were excluded because only imported cases of COVID-19 existed, and no large-scale local transmission occurred in these cities.

#### 2.1.2. Data

**Figure 2:**
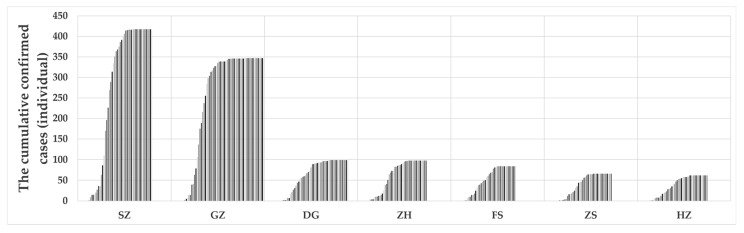
The daily cumulative local confirmed cases in the case study area during the first COVID-19 wave.

Two types of data were used in this study, including COVID-19 confirmed cases and potential influencing factors of the intervention efficiency. The COVID-19 data of the study area were obtained from the Health Commission of each local city and Guangdong province. We chose the first epidemic wave as the corresponding data time window, which refers to the period from seven days before the date when the COVID-19 case was first reported in a city until the date when there had been zero new local cases for the past 14 consecutive days, as shown in Table 1. The spatial distribution of the final cumulative confirmed cases and the daily cumulative confirmed cases of seven cities of Guangdong province are shown in Figure 1 and Figure 2, respectively. Data on potential influencing factors of the intervention efficiency include quantity and quality of medical resources, economic dynamics, and population mobility, which were obtained from the Statistical Yearbooks 2020 of the seven cities, location-based services (LBS) data obtained from open platforms, such as Amap API, and the monthly nighttime light data (https://eogdata.mines.edu/nighttime_light/monthly/v10/2020/) (accessed on 22 April 2022).

### 2.2. Methods

**Figure 3:**
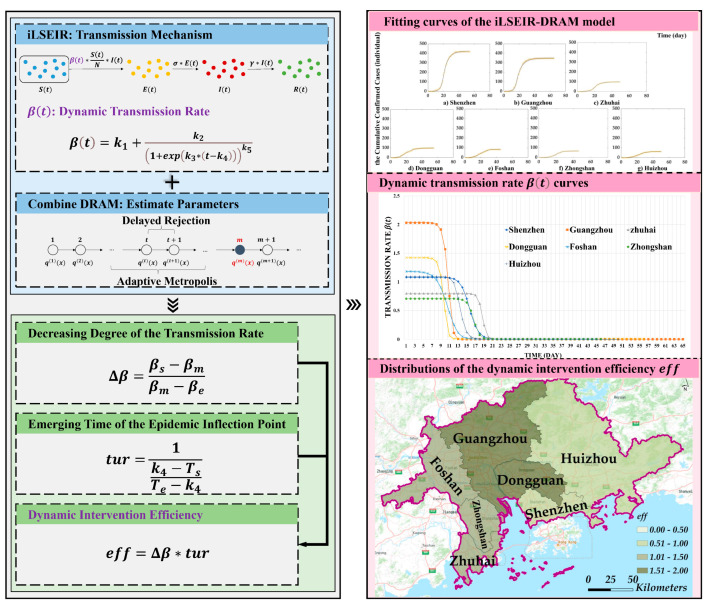
The framework of the scheme for COVID-19 dynamic intervention efficiency evaluation. Here, iLSEIR represents the improved logistic susceptible-exposed-infected-removed (SEIR) model. The DRAM represents the parameter estimation algorithm, which integrated the adaptive metropolis samplers (AM) and the delayed rejection (DR). The abbreviation tur implies the time period ratio between the degree of transmission rate reduction in the infected individuals before and after the fastest decrease. The abbreviation eff is the efficiency of dynamic COVID-19 interventions.

Constructing a model that can well characterize the dynamics of the epidemic transmission process is key to evaluating the efficiency of interventions. In this study, we constructed an iLSEIR-DRAM model to overcome the shortcomings of the existing models, which fail to consider the variation and stochasticity of transmission rate over time. We introduced a five-parameter logistic function to represent the dynamic transmission rate of COVID-19 and estimated the parameters using the DRAM algorithm, which belongs to the adaptive Markov Chain Monto Carlo (MCMC) method. Based on this, we further proposed an indicator to evaluate the efficiency of dynamic interventions and used it to analyze the composite intervention efficiencies of seven cities in Guangdong. The framework of the scheme is shown in Figure 3.

#### 2.2.1. Construction of the Improved LSEIR (iLSEIR) Model

Currently, the SEIR model is the most widely adopted and improved model for simulating epidemics. The original SEIR model divides the population into four groups according to human health statuses (i.e., susceptible (S), exposed (E), infected (I), and removed (R)) and uses a set of differential equations to represent the changes in these subpopulations over time [35,36,37]. There are three important parameters in the SEIR model, namely, the transmission rate β, the incubation rate σ, and the probability of removing from infection γ. They are often used as static parameters to specify the rates of transitions from status S to status E, status E to status I, and status I to status R, respectively [12,37,38].

Due to human interventions, the infection ability of the infected individual will change over time, which means that the transmission rate is time-varying. Thus, assuming it as a fixed value cannot illustrate the actual situation of the epidemic transmission. By introducing a four-parameter logistic function to quantify the time-varying transmission rate β(t), the logistic SEIR (LSEIR) model was proved to be able to simulate the SARS epidemic transmission in Guangzhou in 2003, which experienced three stages, namely, the exponential growth stage, the slowing down stage, and the slow growth stage [30]. According to the white paper published by the State Council Information Office of the People’s Republic of China (http://www.scio.gov.cn/ztk/dtzt/42313/43142/index.htm) (accessed on 11 October 2021), the first wave of COVID-19 epidemic in China has also experienced a similar three-stage epidemic transmission. It is, therefore, possible to construct a new LSEIR-based model to simulate the COVID-19 epidemic transmission.

According to an existing comparative study, the four-parameter logistic function in the original LSEIR model can be considered as a special case of the five-parameter logistic function, which limits it from effectively describing the asymmetrical change in the transmission rate due to the rate differences during the initial fall and the leveling off [39]. Therefore, we constructed an improved LSEIR (iLSEIR) model by using the more general five-parameter logistic function to show the dynamic transmission rate of COVID-19. The ordinary differential equations (ODEs) of the iLSEIR mechanism model are shown in Equations (1)–(6).

(1) $$\frac{dS}{dt}=-\beta \left(t\right)*\frac{S\left(t\right)}{N}*I\left(t\right)$$

(2) $$\frac{dE}{dt}=\beta \left(t\right)*\frac{S\left(t\right)}{N}*I\left(t\right)-\sigma *E\left(t\right)$$

(3) $$\frac{dI}{dt}=\sigma *E\left(t\right)-\gamma *I\left(t\right)$$

(4) $$\frac{dR}{dt}=\gamma *I\left(t\right)$$

(5) $$\beta \left(t\right)=k_{1}+\frac{k_{2}}{{\left(1+exp\left(k_{3}*\left(t-k_{4}\right)\right)\right)}^{k_{5}}}$$

(6) N(t)=S(t)+E(t)+I(t)+R(t)

In Equations (1)–(5), S(t), E(t), I(t), and R(t) represent the population number of susceptible (*S*), exposed (*E*), infected (*I*), and removed (*R*) at time t, respectively. N(t) is the total number of the population in a city. The dynamic transmission rate β(t), which specifically refers to the number of susceptible individuals infected by one infected individual at time t [38], is defined by the five-parameter logistic function in Equation (5). Detailed explanations of the parameters $\left\{k_{1},k_{2},k_{3},k_{4},k_{5},\sigma ,\gamma \right\}$ to be solved are shown in Table 2.

#### 2.2.2. Parameter Estimation and Initialization of the iLSEIR Model

The introduction of the dynamic β(t) to the iLSEIR model involves estimating more parameters. Since the samples obtained from the target probability distribution are based on estimate parameters, they should represent the true distribution, which, however, is unknown and complex. It is, therefore, difficult to sample directly from the target probability distribution. The MCMC is a typical way to solve the problem since it introduces a proposal distribution (i.e., uniform, Gaussian, normal distribution, etc.) to sample indirectly [42,43]. However, it is difficult to determine an effective proposal distribution since non- or slow convergence may occur if it is greatly different from the target distribution. A better strategy is to use an adaptive MCMC algorithm, which can update the proposal distribution continuously [44].

In this study, we used the DRAM algorithm to estimate the parameters. The algorithm optimizes the MCMC by combining adaptive metropolis (AM) samplers and delayed rejection (DR). In the DRAM, the AM algorithm updates the proposal Gaussian distribution continuously and efficiently by using the information so far acquired about the target distribution. The DR considers the rejected information in each time step to preserve the property and reversibility of the Markov Chain lost due to the AM. Herein, the property and reversibility of the Markov Chain refer to the fact that the Markov Chain is a sequence of random samples in which the “next state” (random sample) depends on the “current state” but not on earlier ones [42]. For more information, please refer to Heikki Haario et al.’s series of studies [44,45].

Therefore, the iLSEIR-DRAM model was constructed and then implemented on the Matlab R2020a platform through an external toolbox (https://mjlaine.github.io/mcmcstat/) (accessed on 5 May 2020), mcmcstat. To run the model, we set the initial value of the double boundary range parameters to the median value and the single boundary range parameters to the boundary value (Table 2). Additionally, we performed five restarts on a long Markov Chain to minimize the impact of initial values on epidemiological ODEs [46]. To further reduce the random errors, the average of ten simulations was used as the final results of this study.

#### 2.2.3. Evaluation of the Dynamic Intervention Efficiency

The degree of the decrease in the transmission rate and the emerging time of the epidemic inflection point are important signs of the intervention effects. It is possible to obtain values of these two metrics from the dynamic β(t). This study takes both of them into account to construct a quantitative indicator of the dynamic intervention efficiency. Specifically, we propose the eff to evaluate the efficiency of dynamic COVID-19 interventions by incorporating two sub-indicators Δβ and tur, as shown in Equations (7)–(9). Additionally, the Δβ in Equation (8) indicates the difference in the degree of transmission rate reduction in the infected individuals before and after the fastest decrease in β, which is used to evaluate the reduction in transmission rate. The tur in Equation (9) refers to corresponding time period ratio, which assesses the acceleration degree of the emergence of the epidemic inflection point.

(7) eff=Δβ∗tur

(8) $$\Delta \beta =\frac{\beta_{s}-\beta_{m}}{\beta_{m}-\beta_{e}}$$

(9) $$tur=\frac{1}{\frac{k_{4}-T_{s}}{T_{e}-k_{4}}}$$

**Figure 4:**
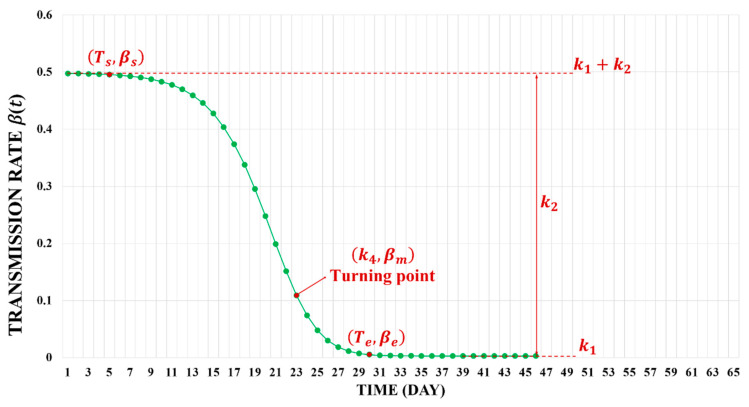
An example of the dynamic transmission rate β(t). In the figure, $k_{1}$ denotes the final transmission rate after the epidemic is under control. $k_{2}$ represents the actual reduction in the transmission rate. $k_{4}$ is the time when the β(t) curve has a turning point. At the turning point, the first order derivative of the β(t) is the largest.

In Equations (7)–(9), $\beta_{s}$ refers to the value of the transmission rate when the β(t) curve starts to fall rapidly (at time $T_{s}$). $\beta_{m}$ is the value of the transmission rate when the β(t) curve falls fastest (at time $k_{4}$). Additionally, $\beta_{e}$ refers to the value of the transmission rate when the β(t) curve tends to be zero (at time $T_{e}$). Considering the states of β(t) at these three time points and important signs of the intervention effects, this paper has constructed an indicator eff. The basis for obtaining $T_{s}$ and $T_{e}$ is that the first order derivative of β(t) is to be within the defined threshold (0.01 or 0.001 is sufficient). That means that between time $T_{s}$ and time $T_{e}$, the effects of interventions cause a significant decrease in the transmission rate. This indicator was used to evaluate the dynamic intervention efficiencies of seven cities in Guangzhou. Figure 4 shows an example curve and its related parameters of dynamic transmission rate β(t), which is useful to understand.

## 3. Results

### 3.1. Simulation Results of the iLSEIR-DRAM

Based on the parameter estimation results, we obtained the mean and median of the average positive rate σ and recovery rate γ, as shown in Table 3. The average incubation period 1/σ and the average time interval from diagnosis to discharge 1/γ can be then inferred accordingly (Table 3). Additionally, the Guangdong Provincial Center for Disease Control and Prevention has estimated a 6.7556-day lag between infection and diagnosis. Therefore, the actual infection period of the COVID-19 patients, from infection to removal, requires adding this time lag. The basic reproductive number $R_{0}$ can be then inferred based on the ratio of the initial transmission rate to the inverse of this time lag. Additionally, 8.02 and 7.40 are the mean and median infections that an index case would cause within a completely susceptible population.

**Figure 5:**
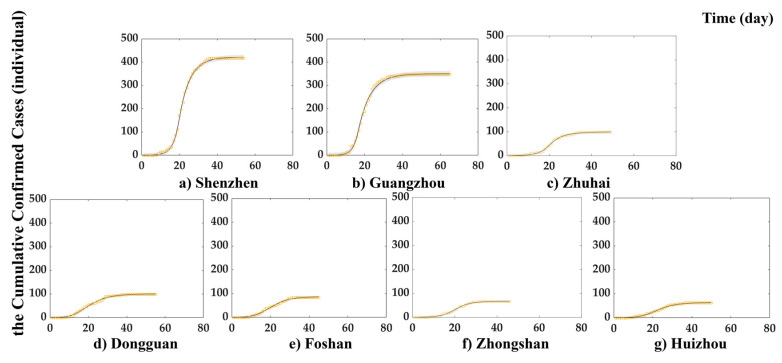
Fitting curves of iLSEIR-DRAM model. The yellow squares are the scatter of actual reported confirmed cases per day. The solid black line represents the simulation results. Gray shading indicates confidence intervals.

Based on $5\times 10^{5}$ times simulations under the 95% confidence level, the cumulative confirmed cases in each city fitted well with determination coefficients $R^{2}$ greater than 0.98 (Table 4). The fitting curves in Figure 5 also prove that the iLSEIR-DRAM model can simulate well in different cities. It indicates that the model with five-parameter logistic function is suitable for representing the time-varying transmission rate of the infected under human intervention.

### 3.2. Dynamic Changes in Transmission Rate Curves

**Figure 6:**
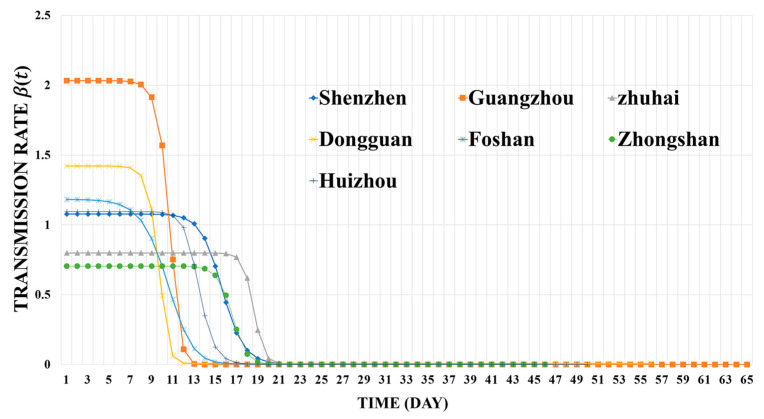
The dynamic transmission rate β(t) curves for each city.

According to the parameter estimation results of in Equation (5), we estimate the dynamic transmission rate β(t) (Figure 6). The seven curves have both similarities and differences. One of the similarities is that all these curves have three-stage changes during the first COVID-19 wave, including the exponential growth stage, the slowing down stage, and the slow growth stage. The second similarity is that the co-effects of various interventions will eventually reduce the daily infectious ability of infected persons to a low level, i.e., the transmission rate β(t) is close to zero, effectively decreasing the risk of COVID-19 infection in the cities.

However, the differences of these transmission rate curves show distinct epidemic processes and intervention efficiencies in each city. Firstly, differences in the maximum and minimum values indicate different transmission rates at the beginning and under control, respectively. As shown in Figure 6, the initial transmission rate was highest in Guangzhou (2.03 person/day), while the lowest was in Zhongshan (0.71 person/day). After the epidemic being under control, the final transmission rates of all the cities were ultimately less than 0.01 person/day. Secondly, policies on COVID-19 might not take effect immediately after implementation due to the latency period. As reflected in the transmission rate curves, differences in the timing of inflection points indicate that it took different amounts of time for each city to bring the COVID-19 epidemic under control. For example, Shenzhen, Zhuhai, Huizhou, and Zhongshan had spent longer time to reach their inflection points. In addition, the curvature at the inflection point of the β(t) curve indicates the speed of intervention effects taken. Curvatures of the seven curves show that the epidemic turns around much faster in Foshan and Guangzhou than in the other five cities.

### 3.3. Results of the Dynamic Intervention Efficiency Evaluation

**Figure 7:**
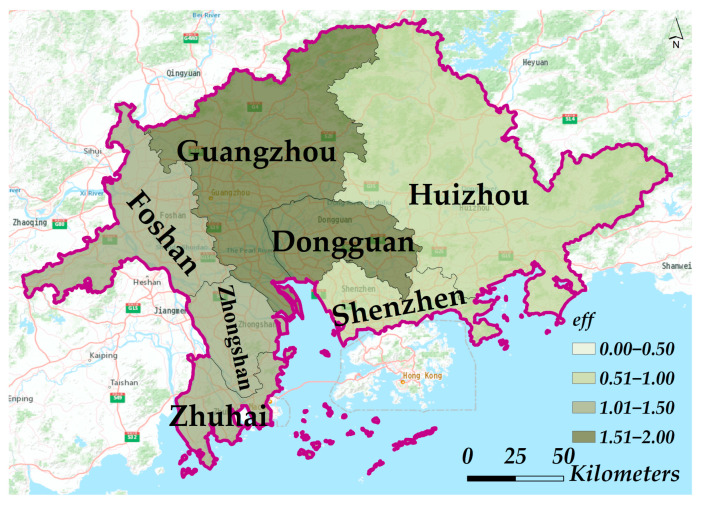
The distributions of the dynamic intervention efficiency eff using the proposed indicator for each city.

As described in Section 3.3, the construction of the indicator considers the intervention efficiency of the COVID-19 epidemic in two aspects, including both the decreasing degree of the transmission rate and the emerging time of the epidemic inflection point. Therefore, the proposed efficiency indicator in Equation (7) is subject to both Δβ in Equation (8) and tur in Equation (9). Based on the parameter estimation results and Equations (7)–(9), the dynamic intervention efficiencies of the cities were rationally evaluated. The integrative evaluation results show that the rank of cities by efficiencies from highest to lowest is Guangzhou, Dongguan, Foshan, Zhongshan, Zhuhai, Shenzhen, and Huizhou (Table 4). Figure 7 gives the spatial distributions of the intervention efficiency in each city.

Table 4 shows that seven cities have different performances in different aspects of epidemic prevention and control. However, the values of Δβ show that significant reduction in transmission rates have taken place in these cities. Consequently, the tur indicates that inflection points appear earlier in Guangzhou, Foshan, and Huizhou than the other four cities. Obviously, since the difference of Δβ is greater than tur, it also contributes more to the efficiency indicator eff. Moreover, the transmission processes of most existing epidemics and EIDs essentially show the three-stage characteristic [47], and the efficiency indicator available in this study are suitable for them.

**Table 4:** The dynamic intervention efficiencies in different cities.

Cities	Determination Coefficient $\left(R^{2}\right)$	Transmission Rate Reduction (Δβ)	Inflection Point (tur)	Efficiency (eff)
Shenzhen	0.9993	0.8464	1.1205	0.9484
Guangzhou	0.9968	5.1795	0.3742	1.9380
Zhuhai	0.9979	1.2223	0.8602	1.0514
Dongguan	0.9955	4.0595	0.4192	1.7019
Foshan	0.9910	1.8904	0.6479	1.2248
Zhongshan	0.9951	1.3863	0.7919	1.0978
Huizhou	0.9860	0.6809	1.3232	0.9010

## 4. Discussion

### 4.1. Analysis of Influencing Factors Related to Efficiency

To better understand the impact of regional socio-economic vulnerability and policy response on dynamic intervention efficiencies, we chose several factors (Table 5) to explore the correlation between these variables and the efficiencies. These socio-economic indicators of the factors were selected or constructed from three aspects: (1) number of medical resources, (2) quality of medical resources, and (3) economic dynamics and population mobility. In addition, we analyzed the relationship of intervention efficiency with the epidemic inflection point and the initial transmission rate, respectively. As shown in Table 5, we first selected the initial transmission rate $\left(\beta_{0}\right)$, the ratio of the time of epidemic turnaround ($E_{IP}$), the size of population ($P_{popu}$), medical resource-related indicators, including the totals of grade A hospitals ($P_{hosp\_a}$), hospital beds ($P_{hosp\_bed}$), doctors ($P_{doc}$), and nurses ($P_{nur}$), and the growth rate of nighttime light values ($P_{npp}$) as possible influencing factors, and then we analyzed the correlation between these factors and the prevention and control efficiency (eff).

**Figure 8:**
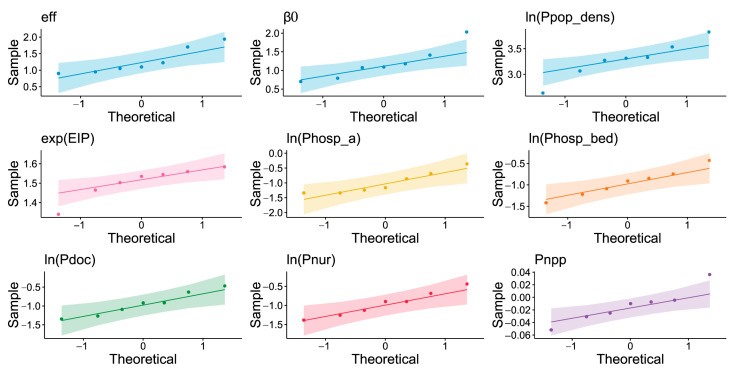
The Q-Q plots of nine variables including eff, $\beta_{0}$, $ln\left(P_{popu}\right)$, $exp\left(E_{IP}\right)$, $ln\left(P_{hosp\_bed}\right)$, $ln\left(P_{doc}\right)$, $ln\left(P_{nur}\right)$, $ln\left(P_{hosp\_a}\right)$, and $P_{npp}$. The scatter point indicates the quantile distribution of the data versus the normal distribution, and the shadow indicates the 95% confidence interval. When the scatter points tend to fall near a straight line, the sample data are close to the normal distribution.

We implemented the Shapiro–Wilk (S–W) normality test to select suitable indicators and their exponential and logarithmic transformations before the correlation analysis, where the two variables involved are required to satisfy a bivariate normal distribution [48]. The results of the S–W test are shown in Table 6. According to the statistic W and P-value, we selected eight indicators $\beta_{0}$, $ln\left(P_{popu}\right)$, $exp\left(E_{IP}\right)$, $ln\left(P_{hosp\_bed}\right)$, $ln\left(P_{doc}\right)$, $ln\left(P_{nur}\right)$, $ln\left(P_{hosp\_a}\right)$, and $P_{npp}$ to perform the Pearson correlation analysis with the eff. In addition, we have drawn Q-Q plots on nine variables, including eff, $\beta_{0}$, $ln\left(P_{popu}\right)$, $exp\left(E_{IP}\right)$, $ln\left(P_{hosp\_bed}\right)$, $ln\left(P_{doc}\right)$, $ln\left(P_{nur}\right)$, $ln\left(P_{hosp\_a}\right)$, and $P_{npp}$ (Figure 8). In Figure 8, the distribution of these scatter points for each Q-Q plot is close to a straight line, which indicates that the original and transformed data used in this study are normally distributed.

**Figure 9:**
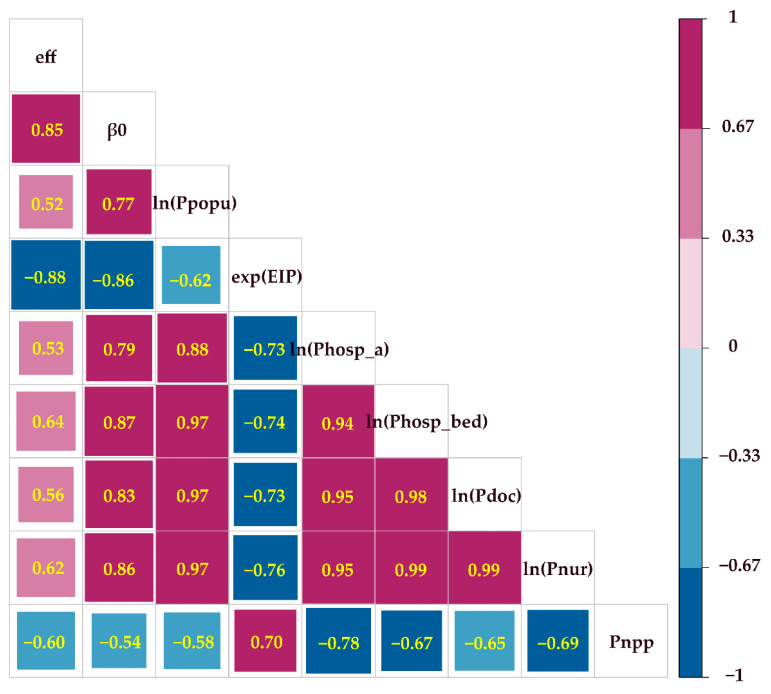
Possible socio-economic factors affected the dynamic intervention efficiency in each city.

As shown in Figure 9, the result of correlation between socio-economic factors and the dynamic intervention efficiency also further validates the rationality of the proposed indicator eff. For example, the efficiency of dynamic COVID-19 interventions (eff) is significantly negatively (−0.88) correlated with the epidemic inflection point ($exp\left(E_{IP}\right)$). That means where interventions are more efficient, the earlier the epidemic inflection point occurs. The population size ($ln\left(P_{popu}\right)$) and the initial transmission rate has a significant positive correlation ($R^{2}$ = 0.77), which indicates that cities with a larger population size will easily have a higher initial transmission rate and face more epidemic pressure.

Allocation of medical resources is another key for the efficiency of dynamic COVID-19 interventions (eff). In general, medical care level is shown in both quantity and quality of medical resources, which are also a city’s foundation to respond to an outbreak of an infectious disease. This study uses the number of hospital beds ($ln\left(P_{hosp\_bed}\right)$), doctors ($ln\left(P_{doc}\right)$), and nurses ($ln\left(P_{nur}\right)$) to indicate the quantity of medical resources and the number of class A hospitals ($ln\left(P_{hosp\_a}\right)$) to indicate the quality of medical resources. Their correlations with eff were 0.64, 0.56, 0.62, and 0.53, with $\beta_{0}$ were 0.87, 0.83, 0.86, and 0.79, and with $exp\left(E_{IP}\right)$ were −0.74, −0.73, −076, and −0.73. This indicates that cities with higher levels of medical care are more effective in preventing and controlling epidemics by reducing the transmission rate and accelerating the inflection point. Therefore, determining how to better allocate medical resources would help patients recover sooner, hence reducing the infection risk [49]. This issue is extremely urgent and challenging.

Additionally, urban, economic, and population activity levels ($P_{npp}$) were significantly and negatively (−0.60) associated with the efficiency of dynamic COVID-19 interventions (eff). As the infection sources of COVID-19 in Guangdong were mainly imported cases from Hubei during the first wave, cities with stronger mobility intensity usually had higher imported risk. Further, the local transmission scale of imported cases is largely influenced by the level of economic and demographic activity within the city. When a city has more restrictions regarding this, the intervention intensity is stronger, and the intervention is more efficient. Because nighttime light data have been shown to reflect this feature well, the growth rate of nighttime light values during February compared to January is used here in this study.

### 4.2. Advantages and Limitations

Although many studies have evaluated the intervention efficiency of COVID-19 through sensitivity analysis of control variables, few of them considered the time-varying transmission rate and evaluated the effectiveness of different policies already applied. In this study, the proposed scheme provides a solution for this issue, which constructed an iLSEIR model by introducing a dynamic transmission rate β(t) and constructed an indicator to realize efficiency evaluation of the interventions that already applied by considering how much the transmission rate reduces and how quickly the epidemic inflection point occurs.

Our method highlights that the transmission rate can be significant in reflecting the effects of human interventions. For most previous COVID-19 studies, the transmission rate was generally set to average, which simplifies its time-varying characteristics. This study represents β(t) using the five-parameter logistic growth function, which intuitively describes the dynamic change in the infectious ability of the infected person under different intervention intensities. Moreover, the function’s three-stage characteristic can explain the effects of interventions on most epidemics, which could provide a reference for other EIDs. Considering seven cities in Guangdong Province as a typical case, we found that the iLSEIR-DRAM model simulated well after introducing the five-parameter logistic growth function, with $R^{2}$ exceeding 0.98 in all the cities. It indicates that the function’s mathematical characteristics well represent the dynamic transmission rate of the infected people under various interventions. Our study also achieves at constructing a quantitative indicator to make the dynamic intervention efficiency comparable in different cities.

This study still has certain limitations. First, this model has only been used to evaluate the efficiency of nonpharmacological interventions. As of 21 March 2022, China had administered about 3.23 billion doses of COVID-19 vaccine, with an 87% full vaccination rate (http://www.nhc.gov.cn/xcs/fkdt/202203/321bbcc05ff548a8bd73d3d31242dc10.shtml) (accessed on 5 August 2022). Although the vaccinated populations are not completely immune, the probability of infection has decreased significantly. Since the effect of the vaccine can also be reflected in the transmission rate, the iLSEIR-DRAM model has the potential to evaluate the co-effects of vaccination and nonpharmacological interventions. Future research may evaluate the efficiency of interventions after the invention of the COVID-19 vaccine. Second, although the five-parameter logistic function is more realistic to represent the dynamic transmission rate β(t), it could not guarantee apparent improvement in the LSEIR model’s simulation accuracy when comparing to the four-parameter logistic function. Future studies should investigate the advantages of four- and five-parameter logistic function used in epidemiology. Third, our work has not performed an attribution analysis for β(t), which is critical to achieve the epidemic prediction. In the future, studies should conduct more in-depth research to address this deficiency.

## 5. Conclusions

Evaluating the intervention efficiency is imperative to optimize prevention and control measures for the outbreak and rapid spread of the COVID-19 epidemic. This study constructed the iLSEIR-DRAM model focusing on the co-effects of all time-varying interventions and quantifying the transmission rate with the five-parameter logistic growth function and has successfully evaluated the COVID-19 dynamic intervention efficiency during the first wave. The proposed model can accurately simulate the transmission process of the COVID-19 epidemic, with the accuracy being over 98% for seven cities of Guangdong. We constructed a quantitative indicator to evaluate the intervention efficiency and analyzed the transmission rate reduction and the inflection point of the COVID-19 epidemic to capture the major influencing factors. The satisfying results of the epidemic transmission simulation and correlation analysis indicate that our proposed scheme can serve as a reference for precise interventions and equal distribution of resources and contribute to responses to other emerging infectious diseases.

## Figures and Tables

**Table 1:** Time range of the first epidemic wave of seven cities in Guangdong province.

Cities	Abbreviation	Beginning of the First Wave	End of the First Wave	Confirmed Cases Number
Shenzhen	SZ	12 January 2020	5 March 2020	417
Guangzhou	GZ	14 January 2020	18 March 2020	347
Dongguan	DG	17 January 2020	12 March 2020	99
Zhuhai	ZH	13 January 2020	1 March 2020	98
Foshan	FS	14 January 2020	27 February 2020	84
Zhongshan	ZS	15 January 2020	29 February 2020	66
Huizhou	HZ	13 January 2020	2 March 2020	62

**Table 2:** Explanations and range setting of main parameters in the iLSEIR epidemiological model.

Parameters	Ranges	Explanation
$k_{1}$	$k_{1}\in \left[0,1\right]$	$k_{1}$ represents the final average transmission rate of infected individuals after implementing interventions.
$k_{2}$	$k_{2}\in [0,+\infty )$	$k_{2}$ represents the variation of the average transmission rate during the entire transmission process. Thus, $k_{1}+k_{2}$ means the initial transmission rate of the infected.
$k_{3}$	$k_{3}\in [0,+\infty )$	$k_{3}$ is the curvature at the inflection point, indicating the speed at which the policy will take effect. The larger $k_{3}$ is the faster the prevention and control effect will appear.
$k_{4}$	$k_{4}\in [0,day)$	$k_{4}$ is the inflection point of the β(t), showing the time lag of the human intervention effect and not exceeding the period to reach half of the maximum cumulative confirmed cases of the local epidemic (day). At the $k_{4}$, the transmission rate drops fastest.
$k_{5}$	$k_{5}\in [0,+\infty )$	$k_{5}$ controls the asymmetry degree of the curve.
σ	σ∈[0,0.5]	σ represents the average positive rate during transmission, which is equal to the inverse of the incubation period. According to Ahmed S. Keshta et al., the COVID-19 incubation period is generally more than 2 days [40]. The range of parameter σ was, therefore, set as [0,0.5].
γ	γ∈[0,1/7]	γ means the probability of removing from infection, which is equal to the reciprocal of the recovery period. For COVID-19 patients, the average treatment time is mostly longer than influenza patients. According to Mahmoud S. Al-Haddad et al., the average duration of illness for the common cold is one week (7 days) [41]; therefore, setting the parameter γ is no more than 1/7 (the reciprocal of 7). The range of parameter γ was, therefore, set as [0,1/7].

**Table 3:** Epidemiological indicators derived from parameter estimation.

Indicators	Average Positive Rate (σ)	Average Incubation Period (1/σ)	Recovery Rate (γ)	Average Time Interval from Diagnosis to Discharge (1/γ)	Recovery Period	Basic Reproductive Number $\left(R_{0}\right)$
Mean	0.27	3.76	0.10	9.72	16.48	8.02
Median	0.28	3.63	0.10	9.53	16.29	7.40

**Table 5:** Possible influencing factors of the intervention efficiency.

Possible Influencing Factors	Explanations
initial transmission rate	$\beta_{0}$: The initial transmission rate in each city, which is equal to $k_{1}+k_{2}$.
population size	$P_{popu}$: The proportion of population size in a given city among these seven cities.
epidemic inflection point	$E_{IP}$: The ratio between the period to reach half of the maximum cumulative confirmed cases of the local epidemic (day) and the duration of the first wave epidemic.
number of medical resources	$P_{hosp\_bed}$: The proportion of hospitals in a given city among these seven cities.
	$P_{doc}$: The proportion of doctors in a given city among these seven cities.
	$P_{nur}$: The proportion of nurses in a given city among these seven cities.
quality of medical resources	$P_{hosp\_a}$: The proportion of grade A hospitals in a given city among these seven cities.
economic dynamics and population mobility	$P_{npp}$: The growth rate of nighttime light values during February compared to January.

**Table 6:** The Shapiro–Wilk (S–W) normality test between eff and eight selected variables. Where the value of statistic W is closer to 1, the significance level *p*-value is greater than 0.05; the hypothesis that the variables obey a bivariate normal distribution is accepted.

Indicators	W	*p*-Value	Pass the Test or Not	Choose the Variable or Not
$\beta_{0}$	0.8945	0.2989	Yes	√
$exp\left(\beta_{0}\right)$	0.7573	0.0151	No	
$ln\left(\beta_{0}\right)$	0.8635	0.1627	Yes	
$P_{popu}$	0.8599	0.1511	Yes	×
$exp\left(P_{popu}\right)$	-	-	-	×
$ln\left(P_{popu}\right)$	0.9139	0.4233	Yes	√
$E_{IP}$	0.8411	0.1017	Yes	×
$exp\left(E_{IP}\right)$	0.8606	0.1533	Yes	√
$ln\left(E_{IP}\right)$	0.7816	0.0268	No	×
$P_{hosp\_bed}$	0.7987	0.0398	No	×
$exp\left(P_{hosp\_bed}\right)$	0.7768	0.0240	No	×
$ln\left(P_{hosp\_bed}\right)$	0.9134	0.4202	Yes	√
$P_{doc}$	0.8374	0.0940	Yes	×
$exp\left(P_{doc}\right)$	0.8223	0.0676	Yes	×
$ln\left(P_{doc}\right)$	0.9019	0.3425	Yes	√
$P_{nur}$	0.8195	0.0636	Yes	×
$exp\left(P_{nur}\right)$	0.7972	0.0385	No	×
$ln\left(P_{nur}\right)$	0.9164	0.4419	Yes	√
$P_{hosp\_a}$	0.7458	0.0115	No	×
$exp\left(P_{hosp\_a}\right)$	0.7230	0.0066	No	×
$ln\left(P_{hosp\_a}\right)$	0.8497	0.1220	Yes	√
$P_{npp}$	0.9455	0.6885	Yes	√
$exp\left(P_{npp}\right)$	0.9389	0.6287	Yes	×
$ln\left(P_{npp}\right)$	-	-	-	×

## Data Availability

Not applicable.
